# The HPV-16 E7 oncoprotein induces centriole multiplication through deregulation of Polo-like kinase 4 expression

**DOI:** 10.1186/1476-4598-10-61

**Published:** 2011-05-24

**Authors:** Nina Korzeniewski, Benjamin Treat, Stefan Duensing

**Affiliations:** 1 Cancer Virology Program, University of Pittsburgh Cancer Institute, Pittsburgh, PA 15213, USA; 2 Molecular Virology and Microbiology Graduate Program, University of Pittsburgh School of Medicine, Pittsburgh, PA 15261, USA; 3 Section of Molecular Urooncology, Department of Urology, University of Heidelberg School of Medicine, 69120 Heidelberg, Germany; 4 Department of Microbiology and Molecular Genetics, University of Pittsburgh School of Medicine, Pittsburgh, PA 15261, USA

## Abstract

**Background:**

Infection with high-risk human papillomaviruses (HPVs) such as HPV-16 is intimately associated with squamous cell carcinomas (SCCs) of the anogenital tract and a subset of oropharyngeal carcinomas. Such lesions, including pre-invasive precursors, frequently show multipolar mitoses and aneuploidy. The high-risk HPV-16-encoded E7 oncoprotein has been shown to rapidly induce centrosome abnormalities thereby causing the formation of supernumerary mitotic spindle poles and increasing the risk for chromosome missegregation. HPV-16 E7 has been found to rapidly induce centriole overduplication, in part, through the simultaneous formation of more than one daughter centriole at single maternal centrioles (centriole multiplication). The precise molecular mechanism that underlies HPV-16 E7-induced centriole multiplication, however, remains poorly understood.

**Findings:**

Here, we show that human keratinocytes engineered to stably express the HPV-16 E7 oncoprotein exhibit aberrant Polo-like kinase 4 (PLK4) protein expression at maternal centrioles. Real-time quantitative reverse transcriptase (qRT-PCR) analysis of these cells revealed an increase of PLK4 mRNA levels compared to control cells. Importantly, the ability of the HPV-16 E7 oncoprotein to induce centriole multiplication was found to correlate with its ability to activate the PLK4 promoter and to up-regulate PLK4 mRNA.

**Conclusions:**

These results highlight the critical role of PLK4 transcriptional deregulation in centriole multiplication in HPV-16 E7-expressing cells. Our findings encourage further experiments to test transcriptional inhibitors or small molecules targeting PLK4 to prevent centriole abnormalities, mitotic infidelity and malignant progression in HPV-associated neoplasms and other tumors in which PLK4 regulation is disrupted.

## Introduction

Infection with high-risk human papillomavirus type 16 (HPV-16) is the leading cause of squamous cell carcinomas (SCCs) of the anogenital tract as well as a subset of oropharyngeal carcinomas [1]. Such neoplasms are commonly genomically unstable and the HPV-16 E7 oncoprotein, together with the E6 oncoprotein, has been shown to play a crucial role in the loss of host cell genome integrity [2].

The HPV-16 E7 oncoprotein disrupts the G_1_/S-phase cell cycle checkpoint on multiple levels to promote unscheduled entry into S-phase and viral genome replication by the host cell DNA replication machinery [3]. High-risk HPV-16 E7 binds and degrades the retinoblastoma tumor suppressor protein (pRB) and inactivates histone deacetylases type -1 and -2 (HDAC-1 and -2) through interaction with Mi2β[4,5]. The HPV-16 E7 oncoprotein has also been shown to interact with transcription factors such as E2F-1 and E2F-6 as well as cyclin/CDK2 complexes [6-9]. Together, these activities not only help to establish a replication-competent milieu in differentiated host keratinocytes but also set the stage for host cellular changes that can promote the progressive loss of genome integrity [10].

Genomic stability is maintained, in part, by the strict control of centriole duplication [11]. Centrioles are the core-forming units of centrosomes, cellular organelles that play a critical role in both cilia and mitotic spindle pole formation [12]. The single centrosome of a non-dividing cell consists of a pair of centrioles, barrel shaped microtubule cylinders, embedded in pericentriolar material [12]. The centrosome duplicates exactly once prior to mitosis in order to form two spindle poles. Deviation from this rule has potentially catastrophic consequences since it can result in supernumerary spindle poles and a defective cell division [13,14]. Centrosome duplication begins in late mitosis/early G_1_-phase of the cell division cycle following centriole separation [15] and recruitment of a protein kinase, polo-like kinase 4 (PLK4), to the wall of the pre-existing, or maternal centrioles, at the site of daughter centriole synthesis [16]. Each maternal centriole serves as a platform for the assembly of normally only one daughter centriole. Centrosome duplication completes during the late-G_2 _phase of the cell division cycle, when the two centriole pairs split to form the mitotic spindle poles.

HPV-16 E7 oncoprotein expression disrupts normal centriole duplication control resulting in the rapid induction of centriole overduplication [17]. This has previously been shown to involve centriole multiplication [18]. This novel pathway is characterized by a single maternal centriole initiating the abnormal simultaneous synthesis of two or more daughter centrioles [18]. Studies in human papillomavirus (HPV)-associated primary human tumors were among the first to demonstrate that centrosome overduplication does in fact occur in human tumors and that the presence of centrosome overduplication correlates with cell division errors [19].

Recently, it was discovered that centriole multiplication involves deregulation of cyclin E/CDK2 complexes, which promote the aberrant recruitment of PLK4 to maternal centrioles [20]. At the same time, however, it was shown that PLK4 protein levels are rate-limiting for centriole multiplication [20]. The present study was therefore designed to examine whether and how the HPV-16 E7 oncoprotein interferes with PLK4 expression to stimulate centriole overduplication.

## Findings and Discussion

### PLK4 is required for HPV-16 E7-induced centriole overduplication and is aberrantly recruited to maternal centrioles

**Figure 1:**
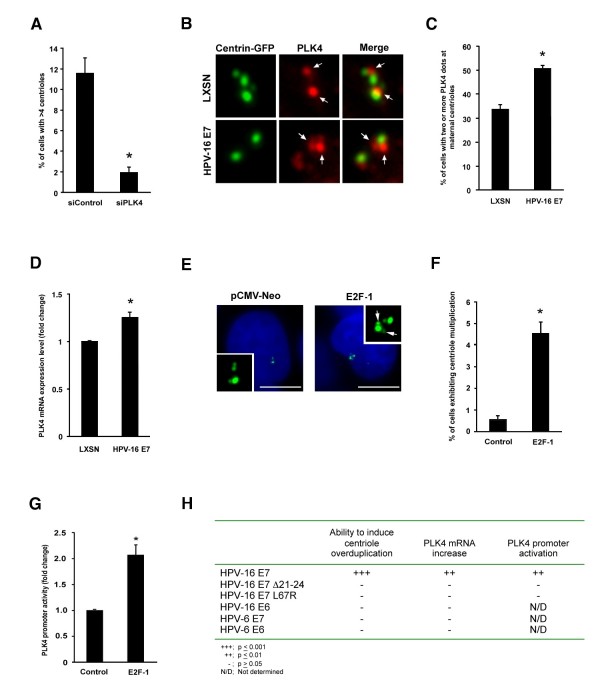
**HPV-16 E7 induces centriole overduplication through transcriptional deregulation and aberrant recruitment of PLK4 to maternal centrioles**. (A) Quantification of centriole overduplication (>4 centrioles per cell) in U-2 OS/centrin-GFP cells transfected with either control (siControl) or PLK4 (siPLK4) siRNA duplexes for 24 h followed by ectopic expression of HPV-16 E7 for another 48 h. Mean and standard error of at least three independent experiments with at least 100 cells counted per experiment are shown. Asterisk indicates statistically significant differences (p ≤ 0.002). (B) Immunofluorescence microscopic analysis of human keratinocytes transduced with either empty vector (LXSN) or HPV-16 E7 for endogenous PLK4 following transiently transfected with centrin-GFP (48 h). Arrows indicate PLK4 dots at maternal centrioles. Note the presence of two dots in a HPV-16 E7-expressing cell (bottom panels). (C) Quantification of the percentage of cells with aberrant (two or more) PLK4 dots at maternal centrioles in human keratinocytes transduced with either a control plasmid, LXSN, or HPV-16 E7. Asterisk indicates statistically significant differences (p ≤ 0.05). (D) Quantitative real-time reverse transcriptase polymerase chain reaction (qRT-PCR) analysis for PLK4 mRNA was performed on total RNA isolated from human keratinocytes expressing either a control plasmid, LXSN, or HPV-16 E7. βactin was used as a control housekeeping gene. Experiments were performed in triplicate and analyzed as described in the Materials and Methods section (Additional File 1). Asterisks indicate statistically significant differences (p ≤ 0.01). (E) Fluorescence microscopic analysis of U-2 OS/centrin-GFP cells transfected with either an empty vector control plasmid (pCMV-Neo) or E2F-1 for 48 h. Nuclei stained with DAPI. Scale bar indicates 10 μm. (F) Quantification of centriole multiplication (>4 centrioles, >1 daughter at one or more maternal centrioles) in U-2 OS/centrin-GFP cells ectopically expressing either a control plasmid or E2F-1 for 48 h. Mean and standard error of two independent experiments with a triplicate of at least 100 cells counted per experiment are shown. Asterisk indicates statistically significant differences (p ≤ 0.002). (G) Quantification of PLK4 promoter activity following transient co-transfection (48 h) of U 2-OS/centrin-GFP cells with the PLK4 promoter construct (pGL3-PLK4) and a transfection control (pRL-CMV) with either an empty vector control (Control) or E2F-1. The bar graph shows fold activation of the PLK4 promoter when compared with the empty luciferase construct, pGL-3, from three independent transfections, and expressed as mean and standard error. Asterisks indicate statistically significant differences (p ≤ 0.001). (H) Table comparing quantification of centriole overduplication (>4 centrioles), PLK4 promoter activation, and PLK4 mRNA upregulation in U-2 OS/centrin-GFP cells ectopically expressing the indicated construct for 48 h and analyzed as described in the Materials and Methods section (Additional File 1).

Depletion of PLK4 protein by small interfering RNA (siRNA) was found to impair the ability of HPV-16 E7 to induce centriole overduplication in U-2 OS/centrin-GFP cells (Figure 1A; Materials and Methods can be found in Additional File 1). The proportion of cells with more than four centrioles was significantly reduced from 11.6% in control siRNA transfected HPV-16 E7 expressing cells to 2% in PLK4-depleted HPV-16 E7 expressing cells (p ≤ 0.002; Figure 1A). This result demonstrates that HPV-16 E7 requires PLK4 to promote centriole multiplication and is in line with previous findings that PLK4 is the rate-limiting factor in this process [20].

We next determined whether PLK4 protein localization is altered in the presence of HPV-16 E7. Non-transformed normal human keratinocytes transduced with either a control plasmid (LXSN) or a plasmid expressing HPV-16 E7 (kindly provided by Susanne Wells, University of Cincinnati, OH, USA) were transiently transfected with a centrin-GFP plasmid (kindly provided by Michel Bornens, Institut Curie, Paris, France [21]) to visualize centrioles followed by immunofluorescence microscopic analysis for endogenous PLK4 (antibody kindly provided by Erich Nigg, University of Basel, Switzerland [22]). In control cells, disengaged centrioles were commonly associated with a single dot of PLK4 localized in close proximity to the site of daughter centriole assembly at the wall of maternal centrioles (Figure 1B, LXSN). In cells stably expressing HPV-16 E7, an increased proportion of cells with two or more dots of endogenous PLK4 at single mothers was detected (Figure 1B, HPV-16 E7). Aberrant PLK4 dots at maternal centrioles were detected in 29 of 86 (33.7%) cells expressing the control plasmid LXSN (Figure 1C). Stable expression of HPV-16 E7 led to a 1.5-fold increase in comparison to controls with aberrant PLK4 dots at maternal centrioles in 45 of 89 cells (50.6%; p ≤ 0.05; Figure 1C).

It is noteworthy that a high percentage of control cells displayed aberrant PLK4 dots at maternal centrioles (33.7%). The frequency of centriole overduplication in control cells was 4% whereas 8.3% of HPV-16 E7-transduced keratinocytes showed centriole overduplication (p ≤ 0.05) while 50.6% of the latter cells showed aberrant PLK4 dots. The discrepancy between aberrant PLK4 recruitment to maternal centrioles and actual centriole overduplication is obvious and in line with a previous report [20]. One reason could be the highly unstable nature of the PLK4 protein [23] but it is also possible that the recruitment of PLK4 is reversible. In addition, it is possible that the target or targets of PLK4 kinase activity may not always be present at effective concentrations to initiate aberrant daughter centriole formation.

### HPV-16 E7 expression up-regulates PLK4 mRNA levels

Since it has previously been reported that PLK4 mRNA levels tightly correlate with PLK4 protein expression, we decided to analyze PLK4 transcription in HPV-16 E7 expressing cells [24,25]. We performed real-time quantitative reverse transcriptase polymerase chain reaction (qRT-PCR) analysis of PLK4 mRNA in human keratinocytes transduced with either a control plasmid (LXSN) or a plasmid expressing HPV-16 E7. We found that stable expression of HPV-16 E7 led to a moderate (1.3-fold) increase of PLK4 mRNA levels in comparison to control cells (p ≤ 0.01; Figure 1D).

HPV-16 E7 oncoprotein expression leads to deregulation of E2F-mediated gene transcription through its chromatin remodeling activities as well as its ability to interact with E2Fs [6,7]. Using a computation-based transcription factor prediction program (TFSearch; http://www.cbrc.jp/research/db/TFSEARCH.html) and analyzing the sequence of the PLK4 gene 1698 bp upstream and 102 bp downstream of the transcription initiation site, we were not able to locate putative E2F-binding sites. However, we did find binding sites for the SP-1 transcription factor within this PLK4 promoter region and SP-1 has been demonstrated to cooperate with E2F-1 to activate gene transcription [26].

Based on the above findings, we transiently overexpressed E2F-1 (plasmid kindly provided by Jacqueline Lees, MIT, Cambridge, MA, USA), in U-2 OS/centrin GFP cells and assayed for centriole multiplication. Overexpression of E2F-1 led to a statistically significant 7.7-fold increase in centriole multiplication from 0.6% in control transfected cells to 4.6% in E2F-1 transfected cells (p ≤ 0.001; Figure 1E and 1F). E2F-1 overexpression was also able to activate the PLK4 promoter. Luciferase activity of a pGL3-based firefly luciferase PLK4 promoter construct (kindly provided by Yi Sun, University of Michigan, Ann Arbor, MI, USA [27]) was found to be enhanced 2.1-fold in the presence of E2F-1 in U-2 OS/centrin GFP cells compared to control transfected cells (p ≤ 0.002; Figure 1G).

Our results show that both the HPV-16 E7 oncoprotein and the transcription factor E2F-1 can stimulate centriole multiplication and that this function correlates with the ability to activate the PLK4 promoter.

### PLK4 promoter activation by HPV-16 E7 correlates with the induction of centriole overduplication

Next, we confirmed that PLK4 mRNA levels were not increased following transient expression of either high-risk HPV-16 E6, low-risk HPV-6 E6 or low-risk HPV-6 E7, none of which mediate centriole overduplication (Figure 1H; [17]). Having determined that up-regulation of PLK4 mRNA was promoted only by high-risk HPV-16 E7 expression, we wanted to explore what functional domains of the E7 oncoprotein were responsible for deregulating PLK4 mRNA levels.

Expression of an HPV-16 E7 mutant with deletion of the amino acid region 21-24, which contains the LCXCE motif (HPV-16 E7 Δ21-24; kindly provided by Karl Münger, The Channing Laboratory, Brigham and Women's Hospital, Harvard Medical School, Boston, MA, USA) and is deficient in both pRB binding and degradation completely lacked the ability to induce centriole overduplication, as has been previously reported (Figure 1H; [28]). Further, an HPV-16 E7 amino acid substitution mutant L67R (HPV-16 E7 L67R; obtained from Karl Münger through Addgene), which is unable to interact with HDACs [4,5], was also unable to induce centriole overduplication to the same extent as wild-type HPV-16 E7 (Figure 1H). This mutant can bind pRB but has a reduced ability to suppress the pRB-induced flat cell phenotype in Saos-2 cells. It has also a reduced protein stability compared to wild-type HPV-16 E7 [4], which makes the interpretation of this result challenging.

We analyzed next whether the ability of the HPV-16 E7 constructs to induce centriole overduplication correlated with their ability to activate the PLK4 promoter and up-regulate PLK4 mRNA. Luciferase activity of the PLK4 promoter construct was enhanced in the presence of wild-type HPV-16 E7 (1.4-fold) but not the HPV-16 E7 mutant constructs (p ≤ 0.01; Figure 1H). In line with the ability of the wild-type HPV-16 E7 oncoprotein to induce PLK4 promoter activation, PLK4 mRNA transcription was increased 1.7-fold following transient expression of wild-type HPV-16 E7 but not the HPV-16 E7 mutant constructs (p ≤ 0.01; Figure 1H). Taken together, these results show an excellent correlation between the ability to induce centriole overduplication and PLK4 promoter activation and mRNA upregulation.

Taken together, our findings depict a mechanism for the rapid induction of centriole multiplication by the HPV-16 E7 oncoprotein. Disruption of the pRB-signaling axis, likely in concert with HDAC-1 and -2 interaction, by HPV-16 E7 promotes the up-regulation of PLK4 mRNA. Deregulation of cyclin E/CDK2 complexes, along with an increase of PLK4 transcription, leads to the aberrant recruitment of excess PLK4 protein to maternal centrioles in the form of multiple PLK4 dots. HPV-16 E7 can interfere with two steps of centriole biogenesis to induce centriole multiplication, namely the increase in PLK4 expression level and the recruitment of PLK4 to maternal centrioles. PLK4 is highly unstable and it is possible that HPV-16 E7 may also interfere with post-translational regulatory mechanisms to increase PLK4 abundance at maternal centrioles.

Support for the important role of gene transcription in HPV-16 E7 induced centriole overduplication comes from a previous study which showed that ongoing RNA polymerase II transcription is necessary for HPV-16 E7 induced centriole overduplication but dispensable for normal centriole duplication [29]. This is in line with findings presented here that up-regulation of PLK4 mRNA transcripts correlates with the ability of HPV-16 E7 to induce centriole multiplication.

Collectively, these results highlight the critical role of PLK4 transcriptional deregulation in centriole multiplication in HPV-16 E7-expressing cells. Our findings encourage further experiments to test transcriptional inhibitors or small molecules targeting PLK4 to prevent centriole abnormalities, mitotic infidelity and malignant progression in HPV-associated neoplasms or other tumors in which PLK4 regulation is found to be disrupted.

## Competing interests

The authors declare that they have no competing interests.

## Authors' contributions

BT performed PLK4 promoter luciferase activity assays. NK performed all other experiments described in this manuscript. NK and SD conceived the project. NK and SD analyzed the results and wrote the manuscript. All authors read and approved the final manuscript.

## Supplementary Material

Additional file 1 **Materials and Methods**. A description of all materials and experimental procedures used in this study. Click here for file
