# Supplementary material for: The HPV-16 E7 oncoprotein induces centriole multiplication through deregulation of Polo-like kinase 4 expression
Source: Mol Cancer. 2011 May 24;10:61. doi: 10.1186/1476-4598-10-61 (PMC3120798; doi:10.1186/1476-4598-10-61)
Supplement: Additional file 1 — Materials and Methods. A description of all materials and experimental procedures used in this study. [file 1476-4598-10-61-S1.DOC]

**ADDITIONAL FILES**

**MATERIALS AND METHODS**

*Cell culture, transfections and inhibitor treatments*

U-2 OS/centrin-GFP cells (centrin-GFP plasmid was kindly provided by Michel Bornens, Institut Curie, Paris, France [1]) were maintained as previously reported [2]. Normal non-transformed human keratinocytes engineered to stably express a control plasmid, LXSN, or HPV-16 E7 (generous gift of Susanne Wells, Cincinnati Children’s Hospital Medical Center, Cincinnati, OH, USA) were maintained in serum-free keratinocyte growth medium (Epilife; Cascade Biologics/Invitrogen) supplemented with human keratinocyte growth supplement (Invitrogen), 50 units/ml penicillin (Cambrex), 50 µg/ml streptomycin (Cambrex), and 0.5 μg/ml fungizone (Invitrogen). For transient transfection of U-2 OS/centrin-GFP cells (48 h), pCMV- or pCDNA3-based plasmids encoding HPV-16 E7, HPV-16 E7 Δ21-24, HPV-16 E7 L67R (kindly provided by Karl Münger, The Channing Laboratory, Brigham and Women’s Hospital, Boston, MA; and by Karl Münger through Addgene [L67R]), HPV-6 E6, HPV-6 E7 (LXSN-based low risk HPV-6 E6 and HPV-6 E7 constructs were kindly provided by Denise Galloway (Fred Hutchinson Cancer Research Center, Seattle, WA, USA) and subcloned into pCMV-based vectors [3]), E2F-1 (kindly provided by Jacqueline Lees, Massachusetts Institute of Technology, Cambridge, MA, USA) or empty vector controls were used and transfected by lipofection (Fugene 6; Roche). For visualization of centrioles in human keratinocytes, cells were transiently transfected (48 h) with centrin-GFP utilizing nucleofection (Amaxa).

*Immunological Methods*

Immunofluorescence staining for PLK4 (mouse monoclonal antibody kindly provided by Erich Nigg, University of Basel, Switzerland) was performed as described previously [2]. Primary antibodies were detected by Rhodamine Red-conjugated secondary antibodies (Jackson Immunoresearch) as previously described [2].

*Small Interfering RNA (siRNA)*

Synthetic RNA duplexes to reduce PLK4 expression were obtained commercially (Ambion) and used according to manufacturer’s protocol.

*Quantitative reverse-transcriptase real-time polymerase chain reaction (qRT-PCR)*

RNA was extracted using the RNase Easy kit (Qiagen). DNase I-treated **t**otal RNA (500 ng) was subjected to qRT-PCR analysis using the one step QuantiTect SYBR Green RT-PCR Master Mix (Qiagen) and the real-time thermocycler iQ5 (Bio-Rad). For qRT-PCR analysis of PLK4 mRNA, the following primer was used: forward 5’-AGTGCTCCCTTTTTCCCAAT-3’ and reverse 5’-AGCAGCACTATGCATGACCA-3’ (147 bp product). The primer sequences for the control housekeeping β-actin used was: forward 5’-TGCGCAGAAAACAAGATGAG-3’ and reverse 5’-CACCTTCACCGTTCCAGTTT-3’ (113 bp product). All reactions were done in triplicate and relative expression of RNAs was calculated using the 2-Delta-Delta-Ct method [4].

*Luciferase Reporter Assay*

A pGL3 firefly luciferase reporter construct expressing an 1.8-kb fragment containing 1698 bp of upstream and 102 bp of downstream sequences around the transcription initiation site of PLK4 was kindly provided by Yi Sun (University of Michigan Comprehensive Cancer Center, Ann Arbor, MI, USA [5]). For determination of PLK4 promoter activation, U-2 OS/centrin-GFP cells were transiently co-transfected by lipofection as stated above with Firefly luciferase reporter construct pGL3-PLK4 or empty vector pGL3-basic as a control (Promega) and wild-type or mutant HPV-16 E7 expression plasmids or the empty pCDNA3-HA vector as a control. Transfection efficiency was normalized by co-transfection with the pRL-CMV plasmid expressing Renilla luciferase (Promega). Luciferase activities were assayed 48 hours after transfection by using the Dual-Glo Reporter Assay System (Promega) according to the manufacturer's instruction and detected in a BioTek Synergy 2 luminometer (BioTek). Results from three independent experiments are expressed as fold-induction after normalization with transfection efficiency and pGL3-Basic control luciferase levels.

*Statistical Methods*

Student’s t test for independent samples was used to calculate statistical significance of centriole counts. Chi-square test was used to determine the statistical significance of aberrant PLK4 recruitment to maternal centrioles.

**REFERENCES**

1. Piel M, Meyer P, Khodjakov A, Rieder CL, Bornens M: **The respective contributions of the mother and daughter centrioles to centrosome activity and behavior in vertebrate cells.** *J Cell Biol* 2000, **149:**317-330.

2. Duensing A, Liu Y, Perdreau SA, Kleylein-Sohn J, Nigg EA, Duensing S: **Centriole overduplication through the concurrent formation of multiple daughter centrioles at single maternal templates.** *Oncogene* 2007, **26:**6280-6288.

3. Spardy N, Duensing A, Charles D, Haines N, Nakahara T, Lambert PF, Duensing S: **The human papillomavirus type 16 E7 oncoprotein activates the Fanconi anemia (FA) pathway and causes accelerated chromosomal instability in FA cells.** *J Virol* 2007, **81:**13265-13270.

4. Livak KJ, Schmittgen TD: **Analysis of relative gene expression data using real-time quantitative PCR and the 2(-Delta Delta C(T)) Method.** *Methods* 2001, **25:**402-408.

5. Li J, Tan M, Li L, Pamarthy D, Lawrence TS, Sun Y: **SAK, a new polo-like kinase, is transcriptionally repressed by p53 and induces apoptosis upon RNAi silencing.** *Neoplasia* 2005, **7:**312-323.
